# Proteome-wide and lysine crotonylation profiling reveals the importance of crotonylation in chrysanthemum (*Dendranthema grandiforum*) under low-temperature

**DOI:** 10.1186/s12864-020-07365-5

**Published:** 2021-01-14

**Authors:** Ping Lin, Hui-ru Bai, Ling He, Qiu-xiang Huang, Qin-han Zeng, Yuan-zhi Pan, Bei-bei Jiang, Fan Zhang, Lei Zhang, Qing-Lin Liu

**Affiliations:** Department of Ornamental Horticulture, Sichuan Agricultural University, 211 Huimin Road, Wenjiang District, Chengdu, Sichuan 611130 People’s Republic of China

**Keywords:** Proteome, Crotonylation, Low-temperature, Chrysanthemum, Biological functions, APX activity

## Abstract

**Background:**

Low-temperature severely affects the growth and development of chrysanthemum which is one kind of ornamental plant well-known and widely used in the world. Lysine crotonylation is a recently identified post-translational modification (PTM) with multiple cellular functions. However, lysine crotonylation under low-temperature stress has not been studied.

**Results:**

Proteome-wide and lysine crotonylation of chrysanthemum at low-temperature was analyzed using TMT (Tandem Mass Tag) labeling, sensitive immuno-precipitation, and high-resolution LC-MS/MS. The results showed that 2017 crotonylation sites were identified in 1199 proteins. Treatment at 4 °C for 24 h and − 4 °C for 4 h resulted in 393 upregulated proteins and 500 downregulated proteins (1.2-fold threshold and *P* < 0.05). Analysis of biological information showed that lysine crotonylation was involved in photosynthesis, ribosomes, and antioxidant systems. The crotonylated proteins and motifs in chrysanthemum were compared with other plants to obtain orthologous proteins and conserved motifs. To further understand how lysine crotonylation at K136 affected APX (ascorbate peroxidase), we performed a site-directed mutation at K136 in APX. Site-directed crotonylation showed that lysine decrotonylation at K136 reduced APX activity, and lysine complete crotonylation at K136 increased APX activity.

**Conclusion:**

In summary, our study comparatively analyzed proteome-wide and crotonylation in chrysanthemum under low-temperature stress and provided insights into the mechanisms of crotonylation in positively regulated APX activity to reduce the oxidative damage caused by low-temperature stress. These data provided an important basis for studying crotonylation to regulate antioxidant enzyme activity in response to low-temperature stress and a new research ideas for chilling-tolerance and freezing-tolerance chrysanthemum molecular breeding.

**Supplementary Information:**

The online version contains supplementary material available at 10.1186/s12864-020-07365-5.

## Background

Plants are often subjected to various environmental stresses that can seriously affect growth and development, including low-temperatures. It has been determined that plants can respond to environmental stresses through a complex set of biological mechanisms [1–5]. Recently, more and more PTMs have shown important roles in plant abiotic stress [6–8]. As the technology of proteomics research has matured, the joint analysis of proteomics and protein modification has been helpful in understanding the mechanism of plant response to environmental stress.

PTMs of proteins include phosphorylation, acetylation, ubiquitination, sumoylation, glycosylation, methylation, and so forth. They are mainly involved in cell activities through signal transduction, the regulation of protein stability and activity, the regulation of gene expression, and the maintenance of genome integrity [9–12]. Among them, the acylation modification that occurs on lysine is the most studied modification [13]. Lysine crotonylation is a new type of histone lysine acylation. For the first time, 28 crotonylation sites were found in the human somatic and mouse male germ cell genomes. Crotonylation modification in histones is closely related to gene transcription and replication. The chemical group modified by crotonylation on histones is crotonyl, with crotonyl-CoA as its main donor [14]. The research in mice has shown that histone crotonylation is associated with acute kidney injury [15]. There are also a large number of crotonylation modifications in non-histone proteins. In non-histone proteins of HeLa cells, 1185 crotonylation modification sites were identified, and they are closely related to DNA and RNA metabolism and cell cycle [16]. A total of 2696 crotonylation sites were identified on 1024 non-histone proteins in H1299 cells. They involve multiple signaling pathways and cell functions, such as RNA processing, nucleic acid metabolism, chromosome assembly, gene expression, and Parkinson’s disease pathways [17]. Current studies have shown that the writers that promote crotonylation are p300 / CBP, CBP, PCAF, and hMOF [17–19]. The erasers for decrotonylation mainly include SIRT1–3, HDAC1, and HDAC3 [17, 20, 21]. Lysine crotonylation in plants was not identified in tobacco until 2017. So far, little research has been done on crotonylation in plants. A total of 2044 and 5995 crotonylation sites were identified in 637 and 2120 proteins of tobacco and papaya, respectively [22, 23]. In rice seedlings, 1265 crotonylation sites were identified on 690 proteins. These modifications are crucial in the regulation of rice gene expression [24]. A total of 45 crotonylation sites were identified in rice histones, which have important functions for rice gene activation under starvation and submergence [25]. In tea leaves, 120 and 151 crotonylated modified proteins were differentially expressed after 3 h and 3 d of ammonium resupply, respectively [26]. These findings suggest that lysine crotonylation may play a potentially important role in plants responding to environmental stress. Lysine crotonylation related to low-temperature stress has not yet been studied.

Chrysanthemum is one of the most important ornamental plants in the cut flower market, and it is susceptible to chilling stress during flowering. Thus, it is important to understand the cold-tolerance mechanism of chrysanthemum and to cultivate new varieties of chrysanthemum. In this study, the dynamic change of proteome-wide crotonylation of chrysanthemum was quantified by TMT labeling and sensitive immuno-precipitation and high-resolution LC-MS/MS. The results showed that 2017 crotonylation sites were identified in 1199 proteins. The crotonylated proteins and motifs in chrysanthemum were compared with tea, rice, papaya, and tobacco to obtain orthologous proteins and conserved motifs. So as to further study the mechanism of crotonylation of chrysanthemums in abiotic stress, we investigated the effects of different crotonylation events on the activity of APX in the antioxidant system through site-directed mutations.

## Results

### Chrysanthemum seedling survival rate and physiological changes under low-temperature treatment

**Fig. 1:**
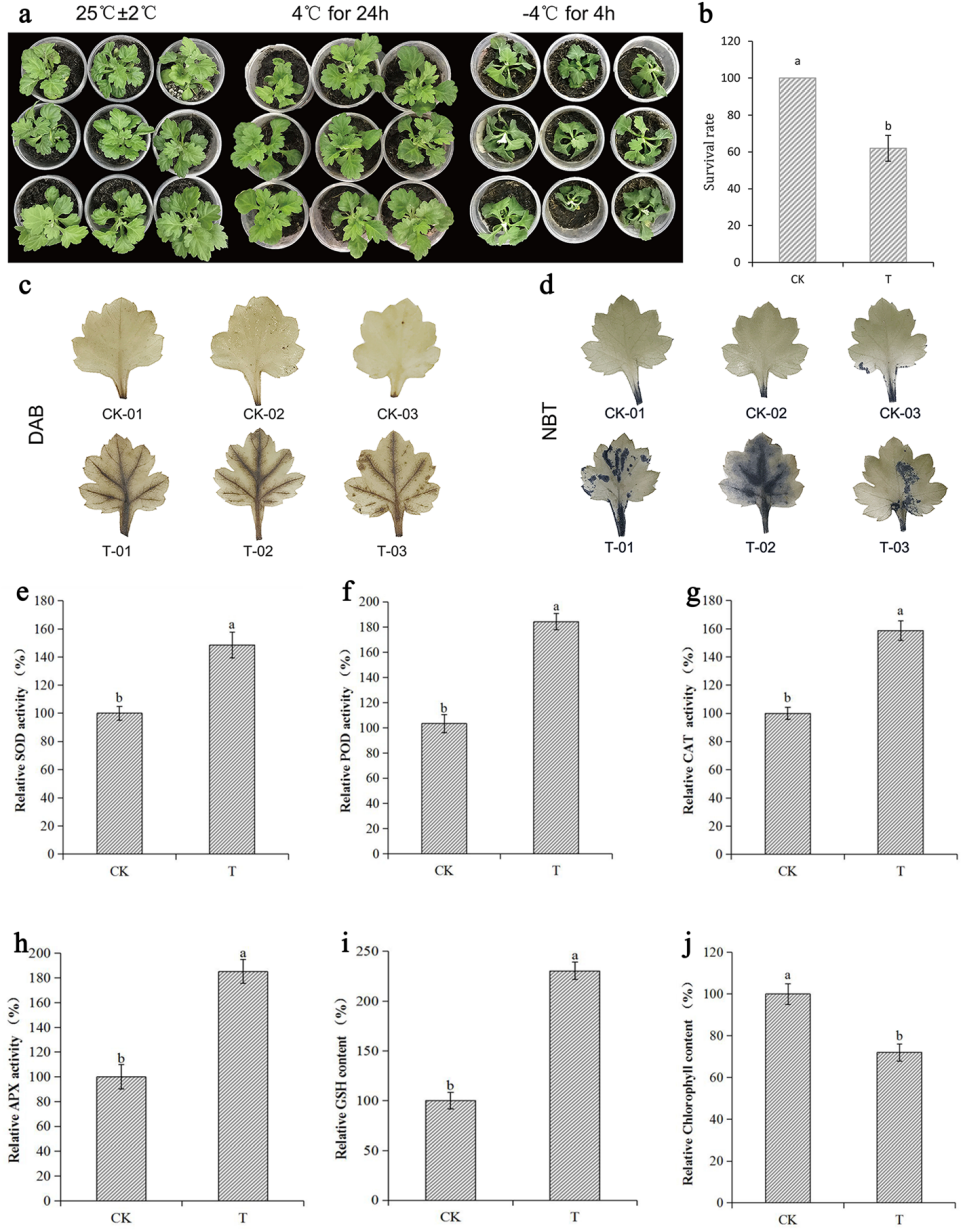
The phenotype, survival rate, and ROS content of chrysanthemum under low-temperature stress. T refers to chrysanthemums treated first at 4 °C for 24 h and then − 4 °C for 4 h, while CK is chrysanthemum without low-temperature treatment. The value measured without low-temperature treatment is taken as 1, and the relative content refers to the ratio between the low-temperature treatment and the untreated. **a** The phenotypic changes of chrysanthemum plants under low-temperature; (**b**) the survival rate of chrysanthemum under low-temperature; (**c**-**d**) histochemical staining with DAB and NBT for assessing the accumulation of H_2_O_2_ and O_2_^−^, respectively, under low-temperature. **e** Relative activity of SOD in chrysanthemum leaves before and after low-temperature treatment; (**f**) relative activity of POD in chrysanthemum leaves before and after low-temperature treatment; (**g**) relative activity of CAT in chrysanthemum leaves before and after low-temperature treatment; (**h**) relative activity of APX in chrysanthemum leaves before and after low-temperature treatment; (**i**) relative content of glutathione in chrysanthemum leaves before and after low-temperature treatment; (**j**) relative content of chlorophyll in chrysanthemum leaves before and after low-temperature treatment. Data represent means and standard errors of three replicates. Different letters above the columns indicate significant (*P* < 0.05) differences according to Duncan’s multiple range test

As shown in Fig. 1a, the chrysanthemum seedlings grew normally, and the phenotype of chrysanthemum did not show noticeable differences from normal circumstances after 24 h treatment at 4 °C. After 4 h treatment at − 4 °C, the leaves of the seedlings appeared wilted and dehydrated, and the whole plant died. Moreover, the survival rate of the control group (CK) was 100% after 2 w of recovery from low-temperature stress, while the treatment group (T) was only 62% (Fig. 1b).

Through histochemical staining of chrysanthemum leaves, we further characterize the oxidative damage to chrysanthemum under low-temperature stress. As shown in Fig. 1c,d, chrysanthemum leaves accumulated more H_2_O_2_ and O_2_^−^ at low-temperature. This indicates that chrysanthemum is under more severe oxidative stress at low-temperature. The activities of antioxidant enzymes (APX, peroxidase (POD), superoxide dismutase (SOD), and catalase isozyme (CAT)) in chrysanthemums treated at low-temperature are significantly higher than those under normal conditions (Fig. 1e-h). The content of glutathione (GSH) is significantly higher than under normal conditions (Fig. 1i). In addition, a decrease in chlorophyll content was also observed under low-temperature (Fig. 1j). These results can prove that chrysanthemum is capable of fighting against the negative effect of low-temperature stress through adjusting the activity of antioxidases.

### Identification of proteome-wide and lysine crotonylation sites in chrysanthemum under low-temperature

This study used a proteomic method based on sensitive immuno-precipitation and high-resolution LC-MS/MS to confirm the crotonylated proteins and their modifcation sites in chrysanthemum (Fig. S1a). The distribution of peptide length in proteomic analysis shows that most peptides were between 7 and 13 amino acids in length, which is consistent with the properties of trypsin peptides (Fig. S1b). At the same time, Fig. S1c shows that the peptide mass error distribution was close to zero, proving the validity of MS data.

A total of 6693 proteins were identified, of which 5339 were quantified. Among the quantified proteins, a total of 393 proteins were upregulated, and 500 proteins were downregulated, including target proteins (1.2-fold threshold; *P* < 0.05). During low-temperature treatment, the number of downregulated proteins was greater than the number of upregulated proteins (Table S1). In addition, the total number of peptide sequences was 2122, the total number of peptides (include modified and non-modified) was 2238, the number of modified peptides was 2173, and the enrichment efficiency was 97.1%. A total of 2017 sites were identified in 1199 proteins, among which 1787 lysine crotonylation sites in 1076 proteins were quantified, and 1089 lysine crotonylation sites in 572 proteins were normalized. The fold-change cutoff was set when proteins with quantitative ratios above 1.2 or below 1/1.2 were deemed significant. Among the quantified proteins after proteome normalization, 89 lysine crotonylation sites in 61 proteins were upregulated and 87 lysine crotonylation sites in 72 proteins were downregulated in the group (Table S2).

We further analyzed the function and characteristics of the identified and quantified proteins by annotating gene ontology, domain pathways, and predicted subcellular localization. Several proteins were found to contain a large number of lysine crotonylation sites in the details of the lysine crotonylation sites and their matching proteins (Table S2). For example, a ‘histone H2B-like’ protein, a ‘probable ATP synthase 24 kDa subunit mitochondrial‘protein, and a ‘stromal 70 kDa heat shock-related, chloroplast-like‘protein contained 14, 14, and 13 crotonylation sites.

### Functional classification analysis of differentially quantified crotonylated proteins in chrysanthemum under low-temperature

**Fig. 2:**
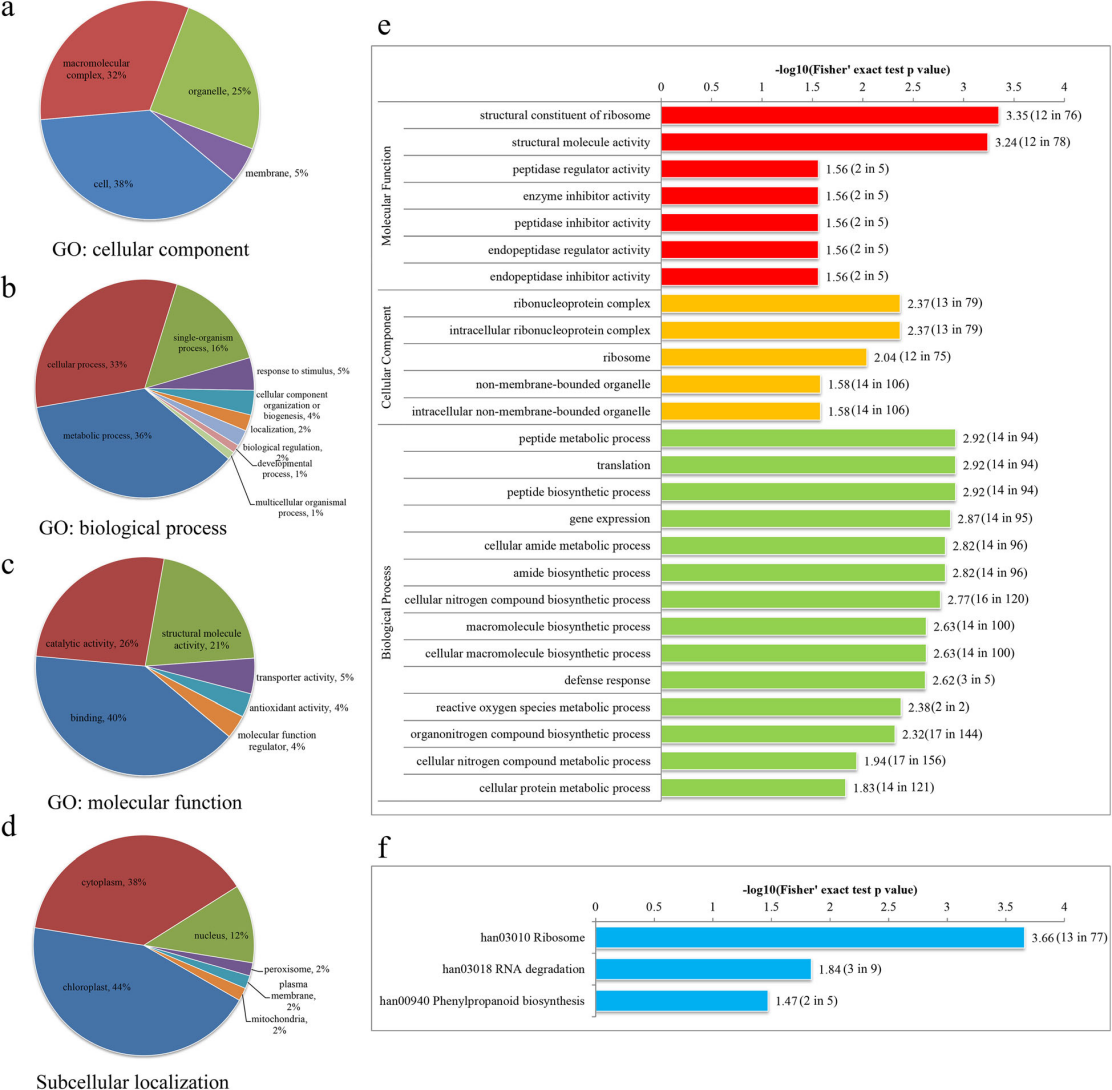
Functional classification and enrichment analysis of differentially quantified crotonylated proteins in chrysanthemum under low-temperature. **a** Cellular component of GO annotation analysis. **b** Biological process of GO annotation analysis. **c** Molecular function of GO annotation analysis. **d** Predicted subcellular localization analysis. **e** GO enrichment analysis. **f** KEGG enrichment analysis. The negative logarithm of Fisher’s exact test *P* value is shown on the X axes. The number of proteins found in each GO class and the number of all proteins present in each GO class were provided in the brackets followed the scores

Crotonylated proteins were annotated by bioinformatics analysis of GO and predicted subcellular localization. The functions of all crotonylated proteins after GO annotations can be divided into three main categories: biological processes, cellular components, and molecular functions. In the cellular component category, the majority of crotonylated proteins were predicted to be related to the cell, the macromolecular complex, the organelle, and the membrane. In the biological process category, many of the crotonylated proteins were enriched in the metabolic process, cellular process, and single-organism process. The analysis of molecular function showed that most crotonylated proteins were related to binding, catalytic activity, structural molecule activity, and transporter activity (Fig. 2a-c). A more detailed classification of differentially quantified crotonylated proteins is shown in Table S3. Predictive analysis of subcellular localization showed that 44% of crotonylated proteins were located in the chloroplast, 38% were located in the cytoplasm, and 12% were located in the nucleus (Fig. 2d).

### Functional enrichment analysis of differentially quantified crotonylated proteins in chrysanthemum under low-temperature

Enrichment analysis of the GO and KEGG pathways was used to further understand the biological function of the crotonylated proteins. GO enrichment analysis showed that, in the molecular function, many crotonylated proteins were significantly enriched in the structural constituent of the ribosome, structural molecule activity, and proton-transporting ATP synthase activity. Based on cellular component enrichment, most crotonylated proteins were mainly enriched in the proton-transporting ATP synthase complex, the catalytic core F, proton-transporting ATP synthase complex, and the large ribosomal subunit. Based on biological process enrichment, most crotonylated proteins were mainly enriched in the cellular nitrogen compound biosynthetic process, the nucleobase-containing compound, and the organonitrogen compound biosynthetic process (Fig. 2e). The KEGG pathway enrichment involved the ribosome and RNA degradation (Fig. 2f). Thirteen crotonylated proteins were only enriched in the ribosome pathway.

### Conservative analysis of crotonylated proteins of chrysanthemum compared with other plants

**Fig. 3:**
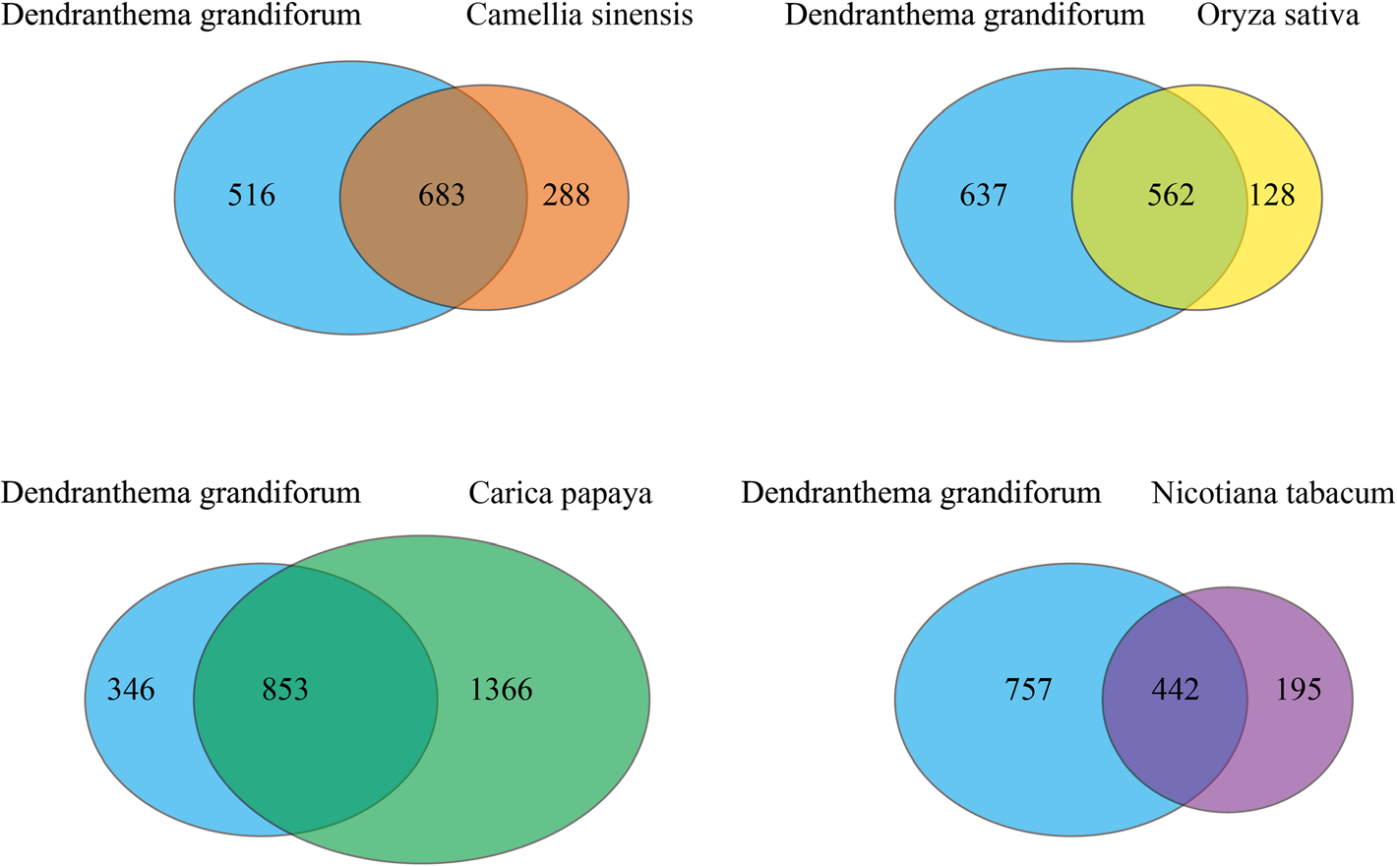
Venn diagram of the orthologous crotonylated protein of chrysanthemum, tea, rice, papaya, and tobacco

We first used BLASTP to compare crotonylated proteins sequences of chrysanthemum(1199) against specified protein sequences, which includes four species: tea(971), rice (690), papaya (2219), and tobacco (637). By applying a reciprocal best BLAST hit approach, we determined the orthologous proteins among these genomes. We found that chrysanthemum has 683, 562, 853, and 442 orthologous crotonylated proteins with camellia, rice, papaya, and tobacco (Fig. 3, Table S4). Meanwhile, among these orthologous crotonylated proteins related to the ribosome pathway, the photosynthesis pathway and the antioxidant system were selected (Table S5–7).

**Fig. 4:**
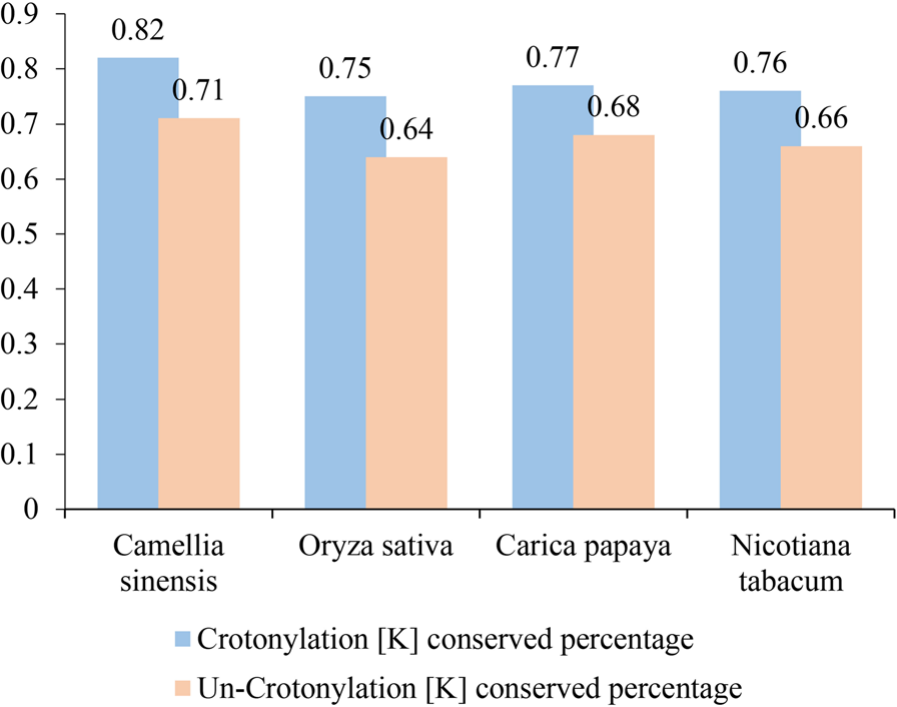
Lysine conservation of chrysanthemum compared with other species

In order to further analyze the orthologous crotonylated proteins of chrysanthemum and other plants, we conducted crotonylated lys conserved analysis. The results showed that chrysanthemum and papaya had the highest number of conserved lysine, and tobacco had the lowest number of conserved lysine (Table S8). However, compared with other plants, the orthologous proteins of chrysanthemum and tea have the highest crotonylated lys conserved percentage and un-crotonylated lys conserved percentage (Fig. 4). This showed that lysine was the most conserved among the orthologous crotonylated proteins of chrysanthemum and tea.

### Motif analysis of lysine crotonylation peptides

**Fig. 5:**
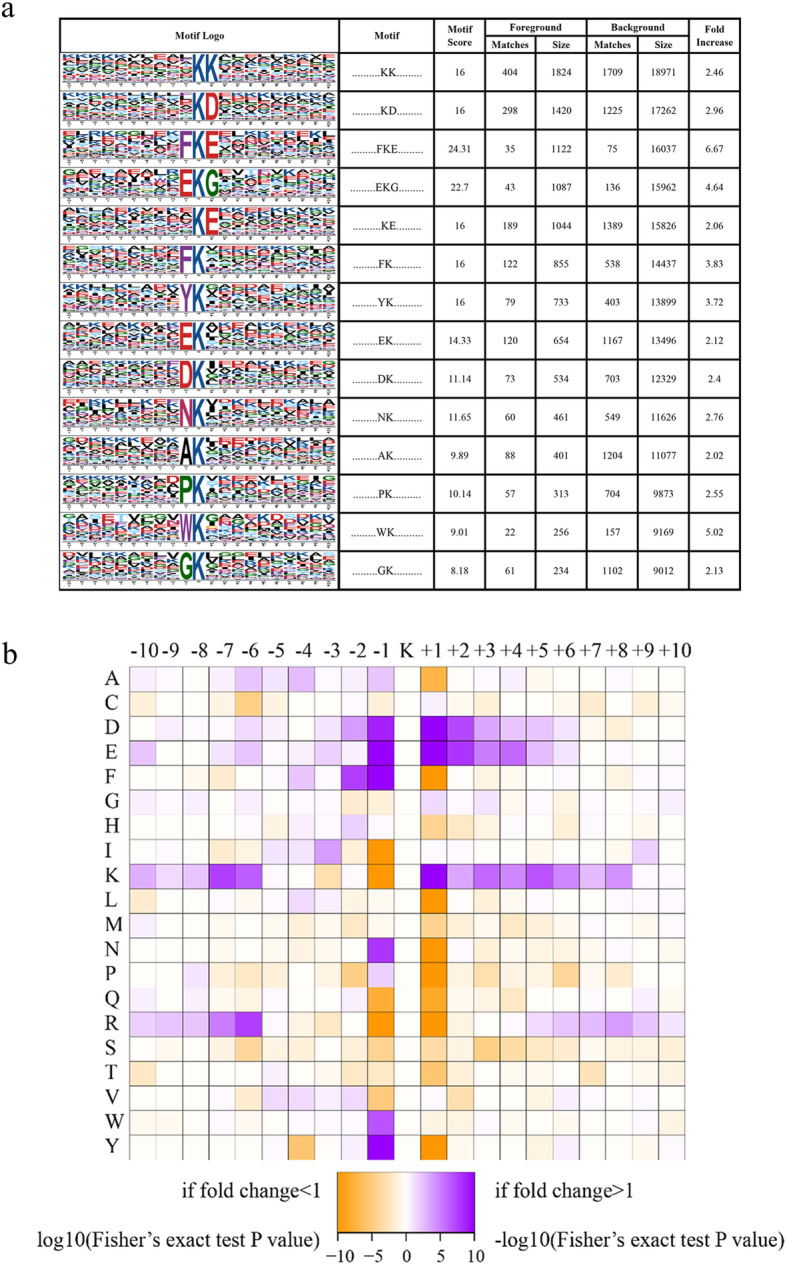
Bioinformatic analysis of lysine crotonylation sites in chrysanthemum under low-temperature. **a** Plot shows the relative abundance of amino acids flanking crotonylated lysine. The relative abundance was counted and schematically represented by an intensity map. The intensity map shows the enrichment of amino acids in specific positions of crotonylated lysine (10 amino acids upstream and downstream of the crotonylation site). **b** Probability sequence motifs of crotonylation sites consisting of 10 residues surrounding the targeted lysine residue using Motif-X

After evaluating the characteristics of all identified crotonylation peptides, it was found that 792 (66.06%) proteins contained a lysine crotonylation site (Kcr site), and 97 (6.59%) proteins contained 4 or more Kcr sites among 1199 Kcr proteins. A total of 14 conserved crotonylation motifs have been identified, which were KcrK, KcrD, FKcrE, EKcrG, KcrE, FKcr, YKcr, EKcr, DKcr, NKcr, AKcr, PKcr, WKcr, and GKcr, and they exhibited different abundances. Analysis of fold increase showed that FKcrE was significantly enriched (Fig. 5a). Positively charged K residues were observed to be enriched at − 10 to − 6 and + 8 to + 1 position, while enrichment of negatively charged residues D and E were observed at positions − 1 to + 5. In accordance with these findings, crotonylation was preferred on lysine residues that were adjacent to aspartic acid, glutamate, and lysine (Fig. 5b).

**Fig. 6:**
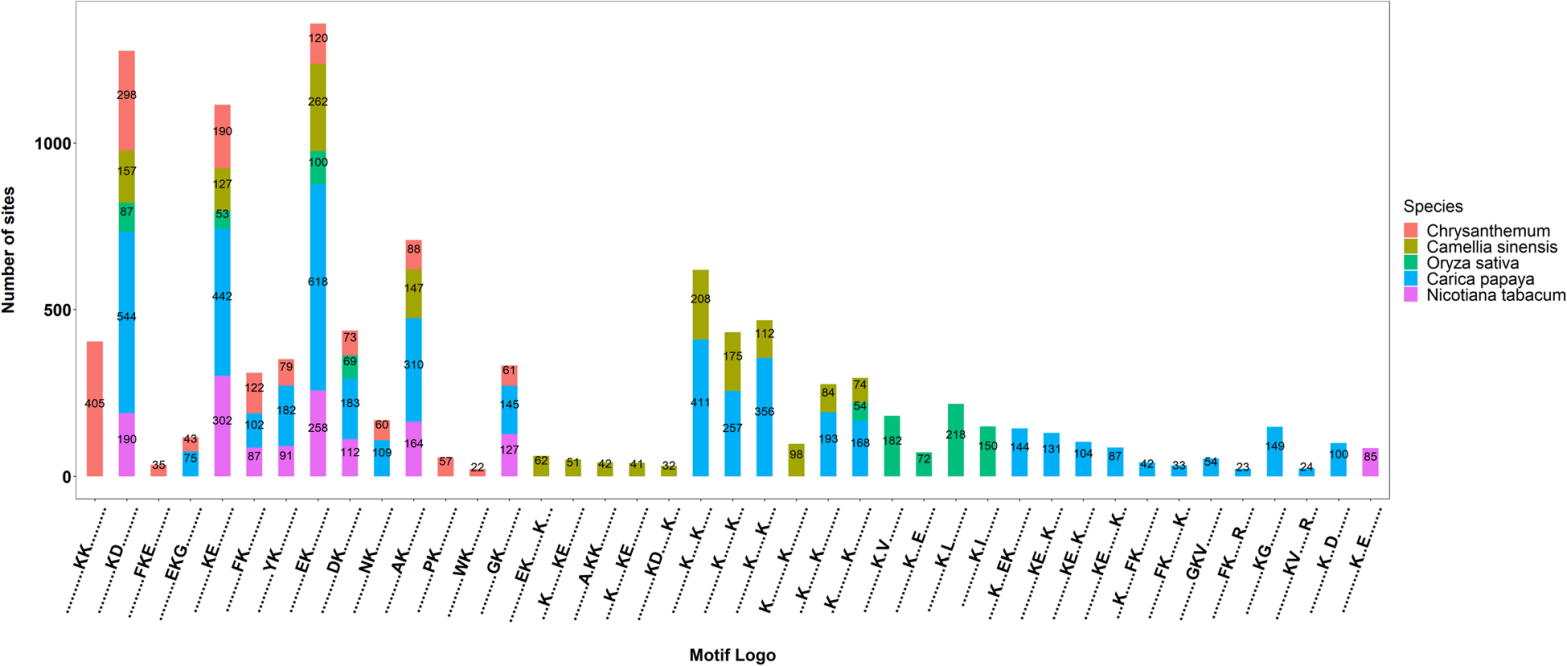
Stacked histogram of crotonylation motifs of chrysanthemum compared with other species

Comparing these conserved motifs identified in chrysanthemums with other plants, a large number of conserved motifs shared with chrysanthemum were found (Fig. 6, Table S9) [22–24, 26]. It is worth noting that chrysanthemum and four other studied plants contain the same conserved KcrD, KcrE, and Ekcr. This indicated that these three motifs are generally conserved in plants. Meanwhile, KcrK, FKcrE, PKcr, and WKcr are new plant crotonylation motifs found in chrysanthemum.

### Crotonylation and proteome crosstalk analysis

A total of 6693 proteins were identified, of which 5339 were quantified. Among the quantified proteins, a total of 393 proteins were upregulated and 500 proteins were downregulated, including target proteins (1.2-fold threshold and *P* < 0.05). In the crotonylation research of TvsCK, there were a total of 2017 lysine crotonylation sites in 1199 proteins, and 1787 lysine crotonylation sites were quantified in 1076 proteins. After the removal of modifications caused by changes in protein levels, 1089 lysine crotonylation sites in 572 proteins were normalized. Among the quantified proteins after proteome normalization, 89 lysine crotonylation sites in 61 proteins were upregulated and 87 lysine crotonylation sites in 72 proteins were downregulated (1.2-fold threshold and *P* < 0.05) (Table S2). In aggregate, most proteins (53) had opposite changes in protein and crotonylation levels, and very few proteins (3) showed consistent changes. A total of 671 proteins were identified in proteome and crotonylation after comparing the proteome and crotonylation datasets.

Meanwhile, according to the correlation analysis between TvsCK’s proteome and crotonylation, there were more points in the 2,4 quadrant than points in the 1,3 quadrant. This suggested that the trends of crotonylation and proteome were not completely consistent, which may be caused by low-temperature treatment (Fig. S2).

### Lysine crotonylation affects APX activity

**Fig. 7:**
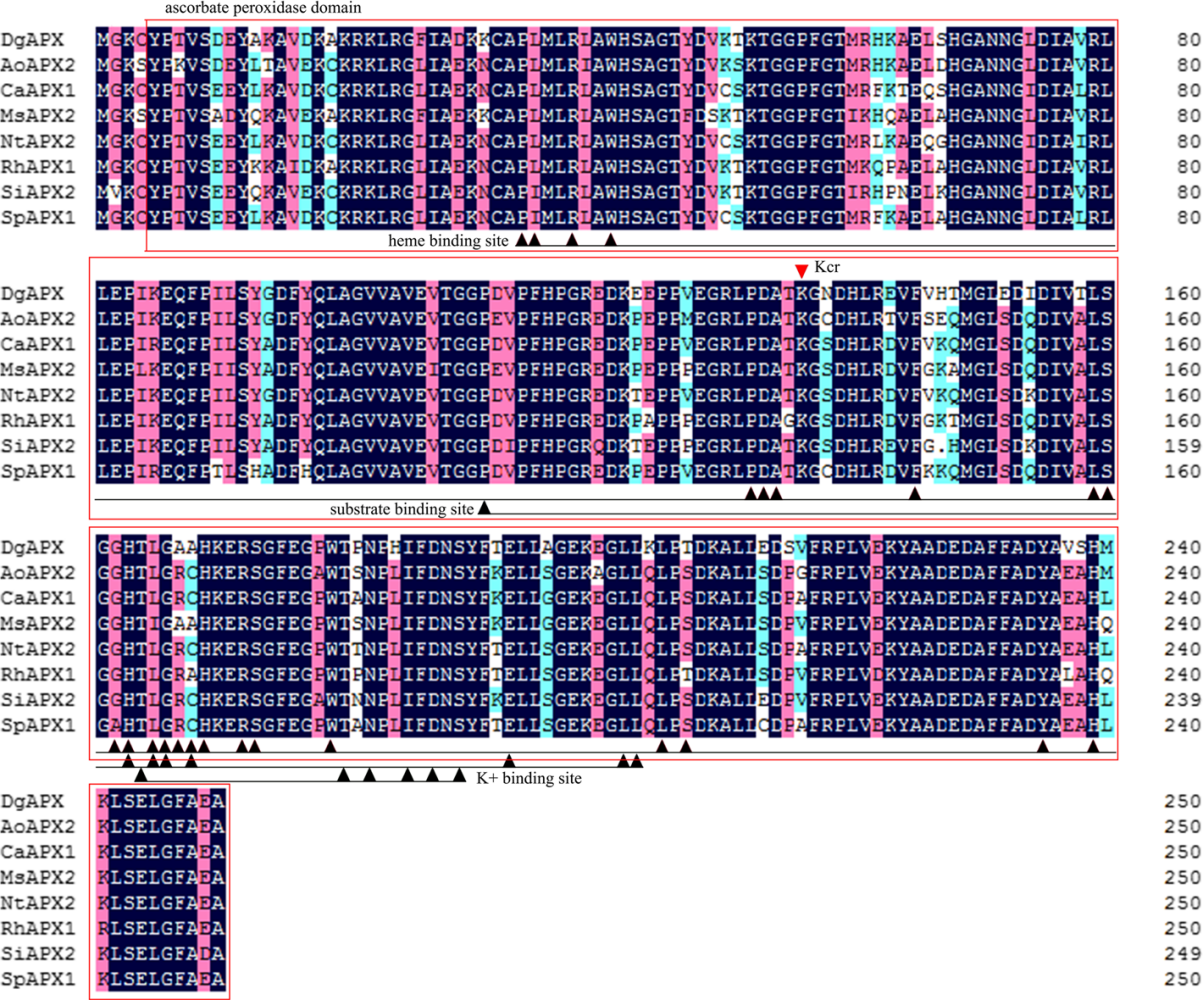
The amino acid sequence of DgAPX was compared with that of other plants. Note: The black triangles indicated the heme binding site, the substrate binding site, and the K^+^ binding site in turn; the red triangles indicated the lysine crotonylation site on the amino acid sequence of DgAPX; the red rectangles indicated the ascorbate peroxidase conserved domain. AoAPX2 (XP_020276942.1) from *Asparagus officinalis*; CaAPX1 (NP_001311967.1) from *Capsicum annuum*; MsAPX2 (AIY27528.1) from *Medicago sativa*; NtAPX2 (NP_001311803.1) from *Nicotiana tabacum*; RhAPX1 (ATP66493.1) from *Rosa hybrid cultivar*; SiAPX2 (NP_001318094.1) from *Solanum lycopersicum*; SpAPX1 (XP_015079739.1) from *Solanum pennellii*

Significant upregulation of crotonylation levels of K136 on APX was detected at low-temperatures (Table S2). APX is an important enzyme for plants to resist ROS. Studies have shown that post-translational modification of proteins can regulate APX activity [27, 28]. However, it has not been reported how lysine crotonylation affects APX activity under low-temperature stress. Based on NCBI (https://www.ncbi.nlm.nih.gov/Structure/cdd/wrpsb.cgi) analysis of the *DgAPX* domain, *DgAPX* contained an ascorbate peroxidase conserved domain consisting of a total of 245 amino acids from Positions 5 to 250, all 24 heme binding sites, all 8 substrate binding sites, and all 6 K^+^ binding sites. Multiple sequence comparisons with other plant APX protein sequences revealed that *DgAPX* was highly identical to other plant APX sequences, and K136 was located in the ascorbate peroxidase domain (Fig. 7).

**Fig. 8:**
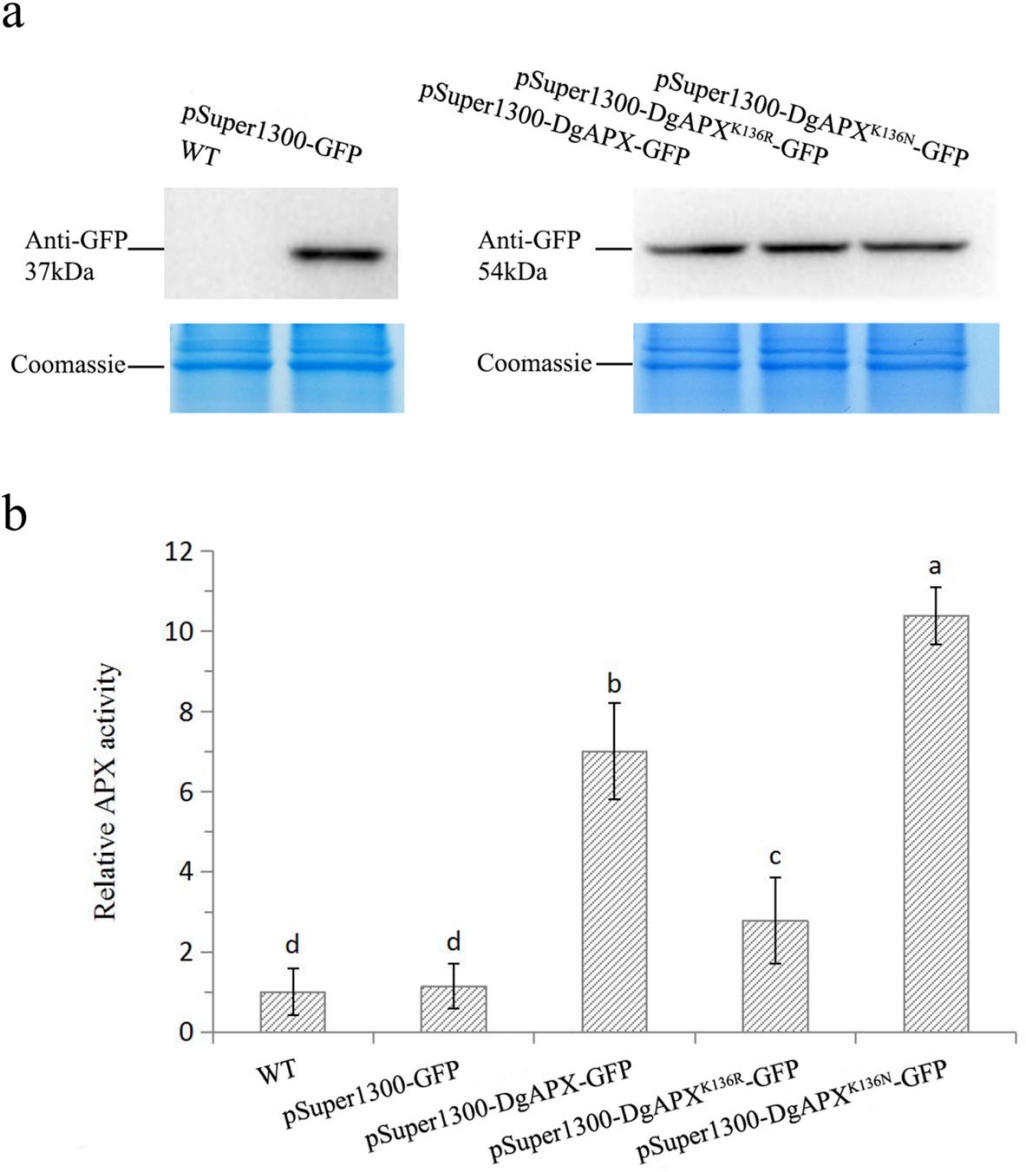
Western blot and APX activity detection. **a** Western blot and coomassie of wildtype tobacco (WT), tobacco infected with empty carrier (pSuper1300-GFP), infected unmutated tobacco (pSuper1300-DgAPX-GFP), infected simulant decrotonylation tobacco (pSuper1300-DgAPX^K136R^-GFP), and infected simulant complete crotonylation tobacco (pSuper1300-DgAPX^K136N^-GFP) under a normal temperature (25 °C; 12 h). **b** APX activity detection of five tobaccos treated under normal temperature (25 °C; 12 h). The different letters above the columns indicate significant (*P* < 0.05) differences according to Duncan’s multiple range test

To further understand how crotonylation at K136 affects APX, we performed a site-directed mutation at K136 (mutated K136 to arginine to simulate decrotonylation and to asparagine to simulate complete crotonylation). We first carried out Western blot and APX activity detection on wild-type tobacco (WT) and tobacco infected with empty carrier (pSuper1300-GFP) under a normal temperature. The results showed that the GFP antibody could only recognize tobacco infected with an empty carrier, while wild-type tobacco could not be identified (Fig. 8a), and there was no significant difference in APX activity between wild-type tobacco and empty carrier tobacco (Fig. 8b). The Western blot results showed that, with consistent protein expression, the APX activity of the simulant complete crotonylation tobacco was 3.38 higher than that of infected unmutated tobacco, while the APX activity of infected unmutated tobacco was 4.23 higher than that of the simulant decrotonylation tobacco. Meanwhile, their APX activity is significantly higher than that of wild-type tobacco and infected tobacco with an empty vector (Fig. 8b).

## Discussion

### Response mechanism of lysine crotonylation and ribosome under low-temperature stress

When plants are subjected to environmental stress, ribosomes may affect protein synthesis. Ribosomes play an important biological role in plant cold tolerance [29]. The ribosomal protein Rpl33 plays an essential role when tobacco is exposed to low-temperature stress [30]. In Arabidopsis under low-temperature stress, ribosomal protein S5 plays a key role. Many proteins related to the low-temperature stress response in rps5–1 are greatly reduced. Overexpression of plastid RPS5 improves cold tolerance of transgenic plants [31]. Rice ribosomal protein TCD11 is involved in the low-temperature response [32]. Under low-temperature stress, rice adapts to environmental changes by inhibiting ribosome biological processes [33]. In tomatoes, low-temperature stress affects the translational extension of ribosomes and prevents plants from repairing damaged proteins [34]. These studies indicate that regulation of ribosomal-related proteins is important for plants to cope with low-temperature stress.

In chrysanthemum, a total of 35 proteins were associated with ribosomes, of which 25 proteins were identified with upregulated crotonylation sites. Fourteen proteins were significantly downregulated at the protein level (Table S10). The crotonylation level of the chaperone-related protein was almost downregulated, while the protein level was not significantly changed. As molecular chaperones, annexin D2-like, annexin D1-like, membrane-associated 30 kDa protein, heat shock cognate 70 kDa protein 2, and temperature-induced lipocalin-1-like assist the protein to fold correctly to perform normal functions. This indicated that the crotonylation modification may play a potential role in biological processes such as ribosomal protein synthesis and processing, and may be related to the plant response to low-temperature stress. The mechanism needs further study.

Interestingly, after comparison with the crotonylation proteins of other plants, among the 35 ribosom-related crotonylated proteins of chrysanthemum, 8 orthologous proteins (22.85%) were also crotonylated in tea, rice, papaya, and tobacco, and 12 (34.28%) were also crotonylated in three of these plants (Table S5). These results suggested that these 20 ribosomal-related proteins were extensively modified by crotonylation in plants. Further research on the crotonylation modification of these proteins will help to understand the potential role of crotonylation in the ribosom pathway.

### Response mechanism of lysine crotonylation and chrysanthemum photosynthesis under low-temperature stress

Abiotic stresses (such as low-temperature, drought, and salt) will lead to the degradation of photosynthesis-related proteins in plants, further reducing plant light energy utilization and being more susceptible to light inhibition [35–37]. In photosynthesis, ribulose 1,5-bisphosphate carboxylase/oxidase (rubisco) is a key enzyme involved in the fixation of CO2 [38]. Rubisco is involved in various physiological responses such as plant growth, photorespiration, glucose metabolism, heat tolerance, low-temperature stress, and salt stress [39–43]. Many studies have shown that the large rubisco subunit is easily degraded under adverse conditions. Under cold stress, 19 protein spots in rice were identified as rubisco large subunit degradation fragments [44]. Drought stress caused significant upregulation of the rubisco subunit degradation products in the beet leaf proteome [45]. Under water stress, the expression level of the large rubisco subunit in black pine was upregulated, and the increase in expression may be due to the degradation of rubisco [46]. The capture and transmission of photosynthetic light energy of higher plants is mainly achieved through the light-harvesting pigment protein complex (LHC I and LHC II), in which LHC II is the most abundant antenna protein in the chloroplast thylakoid of green plants [47]. The chlorophyll a/b-binding protein is a member of the LHC that captures external energy and transmits it to the light system for photosynthesis [48]. Many studies have shown that Photosystem I (PS I) and Photosystem II (PS II) are the important members of photosynthesis in plants. PSI photoinhibition is thought to be the main reason for photosynthetic decline in cold-sensitive plants under low-temperature [49–52]. PS II can maintain photosynthetic efficiency and thus respond to low-temperature stress.

Under low-temperature stress, photosynthesis in chrysanthemum leaves was inhibited, and the chlorophyll content decreased significantly (Fig. 1j). Altogether, 20 crotonylated proteins associated with photosynthesis were identified in chrysanthemum under low-temperature treatment (Table S11). Our results indicated that many proteins related to photosynthesis all showed opposite trends in protein levels and crotonylation modification occurred, such as chlorophyll a/b binding protein, PS I P700 chlorophyll A apoprotein A1, and PS I reaction center subunit II. In addition, 45%(9/20) of the crotonylated proteins are related to ATP synthesis, mainly including ATP synthase subunit b and ATP synthase CF1 alpha chai. Plants photosynthesize mainly through chloroplasts. Chloroplast ATP synthase (CFI and CFO) is present on the chloroplast thylakoid membrane and is a key enzyme for physiological activity in plants. It converts light energy into chemical energy and forms ATP to supply a variety of life activities. A previous study has shown that the large synthesis of the ATP synthase beta subunit improved plant stress tolerance [53]. Most crotonylated proteins associated with photosynthesis contained one crotonylation site. ATP synthase subunit b was crotonylated at four crotonylation sites and modified by upregulation, but none of them caused a significant difference in protein. These results indicated that the crotonylation modification may play a potential role in the photosynthesis pathway, and the change of the crotonylation modification state may be related to the response of photosynthesis-related proteins of plants under low-temperature stress. The mechanism of crotonylation modification in plant photosynthesis pathway under low-temperature stress needs further study.

Interestingly, among the 20 types of crotonylated proteins related to photosynthesis in chrysanthemum, 8 orthologous proteins (40%) were also crotonylated in camellia, rice, papaya, and tobacco, and 6 (30%) were also crotonylated in three of these plants (Table S6). These results suggested that these 14 photosynthesis-related proteins were extensively modified by crotonylation in plants. Further research on the crotonylation modification of these proteins will help to understand the potential role of crotonylation on the photosynthesis pathway.

### Response mechanism of lysine crotonylation and chrysanthemum antioxidant system under low-temperature stress

To prevent oxidative damage caused by low-temperature stress, plants eliminate or reduce excess reactive oxygen species (ROS) or ROS-induced toxic substances through the antioxidant enzyme system [54, 55]. Many proteomic researchers showed that a low-temperature causes a differential expression of antioxidant defense-related proteins [56, 57]. Papaya proteome analysis indicates that lysine crotonylation is involved in the antioxidant system. Altogether, 32 antioxidant-related proteins were identified. Nine of them showed significant changes in crotonylation levels (Table S12). The ROS-related proteins identified from chrysanthemum leaves under low-temperature stress mainly involved POD, CAT, APX, and GST. SOD is an important component of reactive oxygen defense and clearance systems that can disproportionate superoxide anions into molecular oxygen, which has no harmful effect on cells [58, 59]. POD, SOD, CAT, and APX are important components of the active oxygen defense and clearance system. Under abiotic stress including low-temperature, the activities of these antioxidant enzymes will change accordingly. They can help plants clear excess ROS and increase plant resistance [60–63]. GST is also included in the antioxidant enzyme system. GSTs are used to catalyze the combination of glutathione with toxic heterologous or oxidized products, thereby promoting the metabolism of such substances or elimination to reduce oxidative damage [64]. Among the nine antioxidant-related proteins with significant changes in crotonylation levels, five did not have significant changes in protein levels. They are one each of CAT, APX, and SOD and two GSTs. However, the activities of CAT, APX, and SOD increased significantly under low-temperature stress (Fig. 2), which means that crotonylation modification has a potential role on the enzyme activity of the antioxidant system of chrysanthemum and is related to the response of plants under low-temperature stress.

At the same time, our analysis of the orthologous crotonylated proteins of chrysanthemum, tea, rice, papaya, and tobacco showed that, among the 32 antioxidant-related crotonylation proteins of chrysanthemum, there are 9 orthologous proteins (28.12%) were also crotonylated in camellia, rice, papaya, and tobacco, and one (3.12%) was also crotonylated in three of these plants, which includes 6 species: POD, CAT, APX, GST, GPX, and SOD (Table S7). These results suggested that these 10 antioxidant-related proteins were extensively modified by crotonylation in plants. Due to the important role of these antioxidant enzymes in abiotic stress, further studies on the crotonylation modification of these proteins will help to understand the potential role of crotonylation in abiotic stress.

### Lysine crotonylation regulates APX activity in chrysanthemum under low-temperature stress

Among the antioxidant proteins, APX is an important enzyme for plants to resist ROS. APXs can use ascorbic acid as a substrate to convert hydrogen peroxide into water, which is harmless to plants [65, 66]. For example, OsAPX2 can remove ROS from rice and protect rice from low-temperature stress [67, 68]. S-nitrosated residue Cys32 is present in pea APX, and APX can be inactivated or activated by nitration [27]. The sulfide added by S-mercaptolysis can increase the activity of APX in Arabidopsis [28]. These studies have shown that the post-translational modification of proteins can regulate APX activity. Similarly, this study indicated that lysine decrotonylation reduced APX activity and that complete crotonylation increased APX activity (Fig. 8).

Under low-temperature stress, physiological indicators of chrysanthemum showed increased APX activity. Moreover, proteome analysis showed that APX did not significantly downregulate protein levels, but at the level of crotonylation, in site K136, APX was significantly upregulated, which indicated that the lysine crotonylation level of APX was increased under low-temperature stress, and the APX activity increased with the modification level. Crotonylation positively regulated APX activity under low-temperature stress, thereby reducing the oxidative damage caused by low-temperature stress.

## Conclusions

In summary, our study comparatively analyzed proteome-wide crotonylation in chrysanthemum under low-temperature stress, and it further explored all aspects of crotonylation in chrysanthemum’s biological processes, especially the ribosome pathway, photosynthesis, and the antioxidant system. The data provided by this research can be used as an important resource for the analysis of lysine crotonylation functions in chrysanthemum. The crotonylated proteins and motifs in chrysanthemum were compared with tea, rice, papaya, and tobacco to obtain orthologous proteins and conserved motifs. In addition, we identified mutations in specific crotonylation sites and preliminarily explored an important enzyme (APX) regulatory mechanism of the crotonylation anti-oxidation system. Due to the extensive and complex role of crotonylation in cellular processes, how crotonylation participates in the low-temperature threat response requires further research on more specific lysine crotonylation sites to cope with low-temperature stress.

## Methods

### Plant materials and low-temperature treatments

Chrysanthemum (chrysanthemum materials of this study were provided by plant tissue culture room of Sichuan Agricultural Uniersity) was grown on MS medium (200 μL m − 2 s − 1, a 16 h photoperiod, 25 °C/22 °C day/night temperature) for 30 d. It was then transferred to a flowerpot filled with a 1:1 peat and perlite mixture and adapted under the same conditions for 3 d. Leaf samples were taken as CK-01, CK-02, and CK-03 at 0 h of seedling treatment. After low-temperature treatment of seedlings (200 μL m − 2 s − 1, 16 h photoperiod, first at 4 °C for 24 h and then − 4 °C for 4 h) [69], the leaves were sampled as T-01, T-02, and T-03. These two groups of samples were recorded as CK and T and used for histochemical staining and physiological index determination and proteome extraction. For histochemical staining, the reader can refer to Ma ‘s method [70]. Physiological indicators of SOD activity, POD activity, CAT activity, APX activity, GSH content, and chlorophyll content were determined using a kit from Nanjing Jiancheng Bioengineering Institute according to the instructions. The survival rate was calculated after the seedlings were restored for 2 w. The experiment was performed thrice for accuracy.

### Protein preparation, TMT labeling, and HPLC fractionation

Chrysanthemum leaves were ground with liquid nitrogen and lysed in 5 mL of lysis buffer (8 M urea, 2 mM ethylenediaminetetraacetic acid, 10 mM dithiothreitol, and 1% protease inhibitor (Protease Inhibitor CocktailVI, Merck Millipore, USA)) and centrifuged (4 °C, 20,000 g, 10 min) to collect the supernatant. The supernatant was precipitated with 15% trichloroacetic acid (TCA) at − 20 °C for 2 h, and the centrifuged precipitate was washed with cold acetone 3 times and dissolved in buffer (8 M urea, 100 mM Tetraethylammonium bromide (TEAB), pH 8.0) and the catalog product number of BCA was P0011 (Beyotime Biotechnology).

The prepared chrysanthemum protein (700μg) solution was added to 10 mM DL-Dithiothreitol (DTT) and incubated for 1 h at 37 °C, and 10 mM IAM was then added and incubated in the dark for 45 min. Next, the protein was diluted with urea < 2 M. Finally, a mixture with a ratio of trypsin (14 μg) to protein of 1:50 was digested overnight. A mixture with a ratio of trypsin (7 μg) to protein of 1:100 was digested for 4 h. After trypsin digestion, peptide was desalted by a Strata X C18 SPE column (Phenomenex, Strata X C18 SPE column, Los Angeles, USA) and vacuum-dried. Peptide was reconstituted in 0.5 M TEAB. The samples were subjected to 6-fold processing according to the instructions of the TMT kit, and the peptides subjected to TMT were then separated and fractionated by high pH reversed-phase HPLC using an Agilent 300Extend C18 column (5 μm particles, an inner diameter of 4.6 mm, a length of 250 mm, Thermo Scientific, USA). The peptide was separated into 80 fractions using 2–60% acetonitrile (ammonia adjust to pH 10) and combined into 18 fractions. The whole process lasted 80 min, and the peptide was finally dried under vacuum.

### Affinity enrichment

Next, Kcr peptide enrichment was performed. The initial amount of enrichment was 4 mg peptide. Prewashed antibody beads (PTM Biolabs) and tryptic peptides dissolved in NETN buffer (100 mM NaCl, 1 mM EDTA, 50 mM Tris-HCl, 0.5% NP-40, pH 8.0) were gently shaken at 4 °C overnight. The bound peptides were eluted from the beads with 0.1% trifluoroacetic acid (TFA) after the beads were washed four times (25 ul antibody beads, washing volume: 0.5 ml/time, elution volume: 400 ul) with NETN buffer and twice with ddH2O. The eluted fractions were combined and vacuum-dried. The resulting peptides were cleaned with C18 ZipTips (Merck Millipore, ZTC18S960, Billerica, USA) according to the manufacturer’s instructions, followed by LC-MS/MS analysis.

### LC-MS/MS analysis

The above graded peptides were dissolved in 0.1% formic acid (FA) and loaded onto a reversed-phase analysis column (Acclaim PepMap RSLC, 2 μm particles, an inner diameter of 50 μm, a length of 15 cm, Thermo Scientific, USA) for analysis. The gradient was dissolved in 6–22% Solvent B (98% acetonitrile, 0.1% FA v/v) for 19 min, and dissolved in 22–35% Solvent B for 10 min and then Solvent B for 4 min. It was increased to 80% and measured in this state with a constant flow rate of 800 nL/min.

The peptides were subjected to NSI followed by tandem mass spectrometry (MS/MS) in Q ExactiveTM Plus (Q-Exactive Plus, Thermo Scientific, USA) coupled online to the UPLC. The complete peptides were detected at a resolution of 70,000 (NCE setting: 30), and the ion fragments were selected at a resolution of 17,500. A data-dependent procedure that alternated between one MS scan followed by 20 MS/MS scans was applied for the top 20 precursor ions above a threshold intensity greater than 1E4 in an MS survey scan with 30.0 s dynamic exclusion. The electrospray voltage applied was 2.0 kV. Automatic gain control (AGC) was used to prevent overfilling of the orbitrap; 5E4 ions were accumulated for generation of MS/MS spectra (DDA mode (traditional data-dependent collection mode)). For MS scans, the m/z scan range was from 350 to 1800. Fixed first mass was set as 100 m/z. The mass spectrometry proteomics data have been deposited to the ProteomeXchange Consortium via the PRIDE partner repository with the dataset identifier PXD010297.

### Database search

The MS/MS data obtained from the above experiments were analyzed using the Andromeda search engine (v.1.5.2.8), and tandem mass spectra were searched through the Dendrathema grandiflorum database. Peptides (10 ppm) and ion fragments had a mass error of 0.02 Da. Carbamidomethylation on Cys was designated as a fixed modification, and croton substitution of Lys was designated as a variable modification. The false discovery rate (FDR) threshold was set to 1%. Minimum peptide length was set at 7. For quantification, TMT-6-plex was selected. All other parameters in MaxQuant were set to default values. The site localization probability was set as > 0.75.

### Quantitative analysis of protein and lysine Crotonylation

For protein quantification, the ratios of the TMT reporter ion intensities in MS/MS spectra from raw data sets were used to caculate fold changes in the T and CK samples. For each sample, the quantification was mean-normalized at peptide level to center the distribution of quantitative values. Protein quantitation was then calculated as the median ratio of corresponding unique peptides for a given protein. The relative quantitative values of each sample make the data conform to the normal distribution and log2 transform, and the *p* value is then calculated by the two-sample two-tailed T test. When the *p*-value was < 0.05 and the expression ratio was > 1.2, the protein was considered to be upregulated. Conversely, when the p-value was < 0.05 and the expression ratio was < 1/1.2, the protein was considered to be downregulated.

For crontonylation sites quantification, the ratios of the TMT reporter ion intensities in MS/MS spectra from raw data sets were used to caculate fold changes in the T and CK samples. The relative quantitative values of these two samples were the ratio of their quantitative values. In order to remove the modifications caused by changes in protein levels, the proteome was quantitatively normalized, and which means that the ratio of crontonylation sites divided by ratio of corresponding protein in the T and CK samples. When the ratio was greater than 1.2, it was defined as an upward adjustment, and when it was less than 1/1.2, it was defined as an upward adjustment. The resulting crotonylation site on the protein will be used for subsequent biological information analysis.

### Annotation methods

For gene ontology (GO) annotation, we used the UniProt-GOA database (https://www.ebi.ac.uk/GOA/) for annotation. For pathway analysis, we used the Kyoto Encyclopedia of Genes and Genomes (KEGG) database. KEGG annotated results were mapped via Mapper. Predicted subcellular localization of protein analysis was performed by WolfPsort software. We also analyzed eukaryotic sequences using the new version of PSORT/PSORT II to complete the analysis. In order to analyze the enrichment of identified proteins relative to the differentially expressed protein against all identified proteins, a two-tailed Fisher’s test was used. Calibration of multiple hypothesis tests was performed using standard error discovery rate control methods. It was considered significant when the *p*-value of the enriched cluster was < 0.05.

Ten amino acids above and below specific positions in all identified protein sequences were used as analysis objects, and they were analyzed by Motif-X software [71]. All database protein sequences were used as background database parameters, and other parameters used the default.

### Conservative analysis

To determine the degree of evolutionary conservation of crotonylation, we first used BLASTP to compare crotonylated protein sequences of Dendranthema grandiforum (PXD010297) against specified protein sequences, which includes 4 species: *Camellia sinensis* (PXD011610), *Oryza sativa* (PXD008716), *Carica papaya* (PXD008166), and *Nicotiana tabacum* (IPX0000889000). By applying a reciprocal best BLAST hit approach, we determined the orthologous proteins among these genomes. For each orthologous group, we used MUSCLE v3.8.31 to perform multiple sequence alignment. We then determined the lysine conservation for each species by counting the total number of conserved crotonylated lysine, and the total number of conserved non-crotonylated lysine was considered to be conserved if both the *Dendranthema morifolium* protein and the query protein in the multiple sequence alignment were lysine residues at the aligned positions. All lysine residues of the proteins identified in this study were considered as controls. Mean conservation of the crotonylated and control Lys between *Dendranthema morifolium* sequences and sequences from other microorganisms in the specified protein sequences were plotted separately. *P*-values were calculated for each comparison using Fisher’s exact test.

### Site-directed mutagenesis and vector construction

The conserved domain analysis of DgAPX was performed in NCBI (https://www.ncbi.nlm.nih.gov/Structure/cdd/wrpsb.cgi). The amino acid sequence of DgAPX was compared with the APX amino acid sequence of other plants by DNAMAN. L-ascorbate peroxidase (APX) was subjected to whole-gene synthesis, and APX site K136 was mutated to arginine (R) and asparagine (N). We simulated the complete crotonylation (K136N) and complete de-crotonylation (K136R) according to the mutated charge stability. Two mutants and one no-mutant were selected by DNA sequencing. These three sequences were constructed on the expression vector (pSuper1300-GFP), respectively. Gene synthesis and expression vector construction were completed by Sangon Biotech (Shanghai) Co., Ltd.

### Expression of recombinant APX

Three constructs (pSuper1300-DgAPX-GFP, pSuper1300-DgAPXK136R-GFP, and pSuper1300-D-gAPXK136N-GFP) and empty pSuper1300-GFP were, respectively, transformed into Agrobacteriu-m GV3101 cells. The cells were cultured in Luria–Bertani (LB) medium (100 mg/L kanamycin, 100 mM MES, 40 μM AS) until the OD600 reached 0.8. The cells were centrifuged(4000 rpm, 10 min, 25 °C) and collected, and the cells were resuspended in an equal volume of buffer (10 mM MgCl2、200 μM AS). After being in the dark for 4 h, it was immersed in the leaves of N. benthamiana and co-cultured for 48 h. The infected tobacco and uninfected tobacco were treated at a normal temperature (25 °C; 12 h). Tobacco leaves were sampled for Western blot and enzyme assay.

### Western blot and enzyme assay

Protein extraction was performed, and concentration was determined for each sample as before. The prepared protein samples were separated on a 15% SDS-PAGE gel and transferred to a polyvinylidene fluoride fluoropolymer (PVDF) membrane (0.22 μm, Millipore Cat. No: ISEQ00010). The membrane was then blocked with TBST containing 5% Skim Milk for 1 h. ProteinFindTM Anti-GFP Mouse Monoclonal Antibody was used in the Western blot analysis. Detection was performed with Horseradish Peroxidase-conjugated Anti-Mous IgG(H + L) secondary antibody and EasySee® Western Blot Kit. APX activity of different samples was measured using an ascorbate peroxidase activity assay kit of Nanjing Jiancheng Bioengineering Institute. The method was performed according to the instructions.

## Supplementary Information

**Additional file 1: Fig. S1**. Experimental strategy and the basic information of all identified peptides.

**Additional file 2: Fig. S2**. Crotonylation and proteome relationship quantification.

**Additional file 3: Table S1**. Maxquant files for protein identification and quantification.

**Additional file 4: Table S2**. Maxquant files for lysine crotonylated identification and quantification.

**Additional file 5: Table S3**. Segmentation results of GO annotation.

**Additional file 6: Table S4**. Orthologous crotonylated protein of chrysanthemum, tea, rice, papaya and tobacco.

**Additional file 7: Table S5**. Ribosome-related orthologous crotonylated proteins of chrysanthemum compared with other species.

**Additional file 8: Table S6**. Photosynthesis-related orthologous crotonylated proteins of chrysanthemum compared with other species.

**Additional file 9: Table S7**. Antioxidant enzyme-related orthologous crotonylated proteins of chrysanthemum compared with other species.

**Additional file 10: Table S8**. Lysine Conservation of chrysanthemum compared with other species.

**Additional file 11: Table S9**. crotonylation motifs of chrysanthemum compared with other species.

**Additional file 12: Table S10**. Ribosome-related proteins and crotonylation sites in chrysanthemum.

**Additional file 13: Table S11**. Photosynthesis-related proteins and crotonylation sites in chrysanthemum.

**Additional file 14: Table S12**. Antioxidant enzyme-related protein and crotonylation site in chrysanthemum.

**Additional file 15: Supplementary Figure 3**. Original image of coomassie in wildtype tobacco (WT), tobacco infected with empty carrier (pSuper1300-GFP) showed in Fig. 8a. **Supplementary Fig. 4**. Original image of coomassie in tobacco infected unmutated tobacco (pSuper1300-DgAPX-GFP), infected simulant decrotonylation tobacco (pSuper1300-DgAPXK136R-GFP), and infected simulant complete crotonylation tobacco (pSuper1300-DgAPXK136N-GFP) showed in Fig. 8a. **Supplementary Fig. 5**. Original image of western blot in wildtype tobacco (WT), tobacco infected with empty carrier (pSuper1300-GFP) showed in Fig. 8a. **Supplementary Fig. 6**. Original image of western blot in tobacco infected unmutated tobacco (pSuper1300-DgAPX-GFP), infected simulant decrotonylation tobacco (pSuper1300-DgAPXK136R-GFP), and infected simulant complete crotonylation tobacco (pSuper1300-DgAPXK136N-GFP) showed in Fig. 8a.

## Data Availability

The main datasets of Dendranthema grandiforum PXD010297 (ProteomeXchange dataset PXD010297), *Camellia sinensis* (ProteomeXchange dataset PXD011610), *Oryza sativa* (ProteomeXchange dataset PXD008716), *Carica papaya* (ProteomeXchange dataset PXD008166), and *Nicotiana tabacum* (iProX dataset IPX0000889000) used in this study are available at http://proteomecentral.proteomexchange.org/cgi/GetDataset?ID=PXD010297, http://proteomecentral.proteomexchange.org/cgi/GetDataset?ID=PXD011610, http://proteomecentral.proteomexchange.org/cgi/GetDataset?ID=PXD008716, http://proteomecentral.proteomexchange.org/cgi/GetDataset?ID=PXD008166, and https://www.iprox.org//page/project.html?id=IPX0000889000.

## Abbreviations

APX: Ascorbate peroxidase
CAT: Catalase isozyme
GSH: Glutathione
GST: Glutathione S-transferase
GPX: Glutathione peroxidase
GO: Gene ontology
KEGG: Kyoto encyclopedia of genes and genomes
PTM: Post-translational modification
POD: Peroxidase
SOD: Superoxide dismutase
