# Supplementary figures and images for: Proteome-wide and lysine crotonylation profiling reveals the importance of crotonylation in chrysanthemum (Dendranthema grandiforum) under low-temperature
Source: BMC Genomics. 2021 Jan 14;22:51. doi: 10.1186/s12864-020-07365-5 (PMC7809856; doi:10.1186/s12864-020-07365-5)

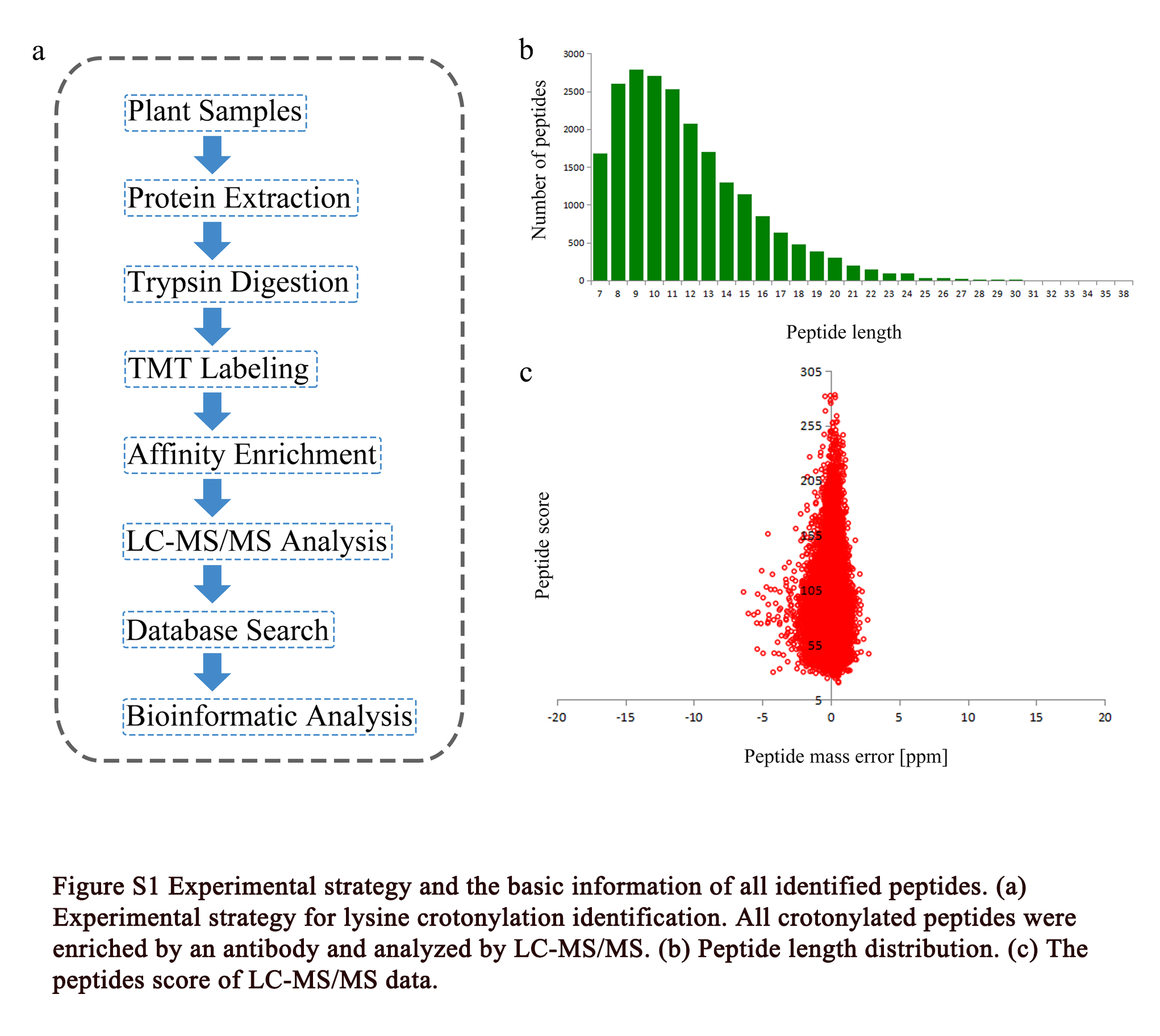

Supplement: Supplementary file 1 — Additional file 1: Fig. S1. Experimental strategy and the basic information of all identified peptides. [file 12864_2020_7365_MOESM1_ESM.tif]

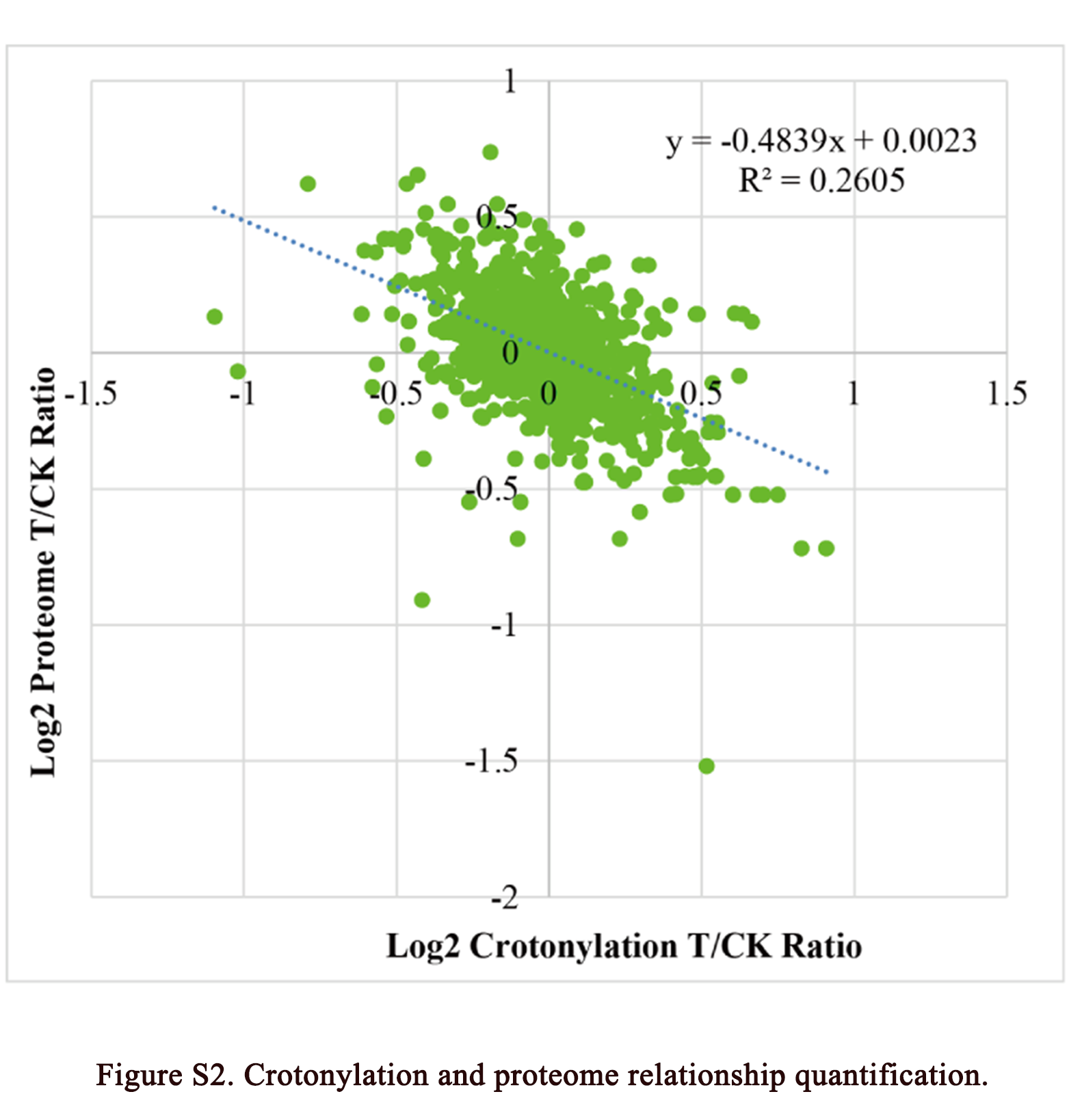

Supplement: Supplementary file 2 — Additional file 2: Fig. S2. Crotonylation and proteome relationship quantification. [file 12864_2020_7365_MOESM2_ESM.tif]
