# Supplementary material for: Proteome-wide and lysine crotonylation profiling reveals the importance of crotonylation in chrysanthemum (Dendranthema grandiforum) under low-temperature
Source: BMC Genomics. 2021 Jan 14;22:51. doi: 10.1186/s12864-020-07365-5 (PMC7809856; doi:10.1186/s12864-020-07365-5)
Supplement: Supplementary file 15 — Additional file 15: Supplementary Figure 3. Original image of coomassie in wildtype tobacco (WT), tobacco infected with empty carrier (pSuper1300-GFP) showed in Fig. 8a. Supplementary Fig. 4. Original image of coomassie in tobacco infected unmutated tobacco (pSuper1300-DgAPX-GFP), infected simulant decrotonylation tobacco (pSuper1300-DgAPXK136R-GFP), and infected simulant complete crotonylation tobacco (pSuper1300-DgAPXK136N-GFP) showed in Fig. 8a. Supplementary Fig. 5. Original image of western blot in wildtype tobacco (WT), tobacco infected with empty carrier (pSuper1300-GFP) showed in Fig. 8a. Supplementary Fig. 6. Original image of western blot in tobacco infected unmutated tobacco (pSuper1300-DgAPX-GFP), infected simulant decrotonylation tobacco (pSuper1300-DgAPXK136R-GFP), and infected simulant complete crotonylation tobacco (pSuper1300-DgAPXK136N-GFP) showed in Fig. 8a. [file 12864_2020_7365_MOESM15_ESM.docx]

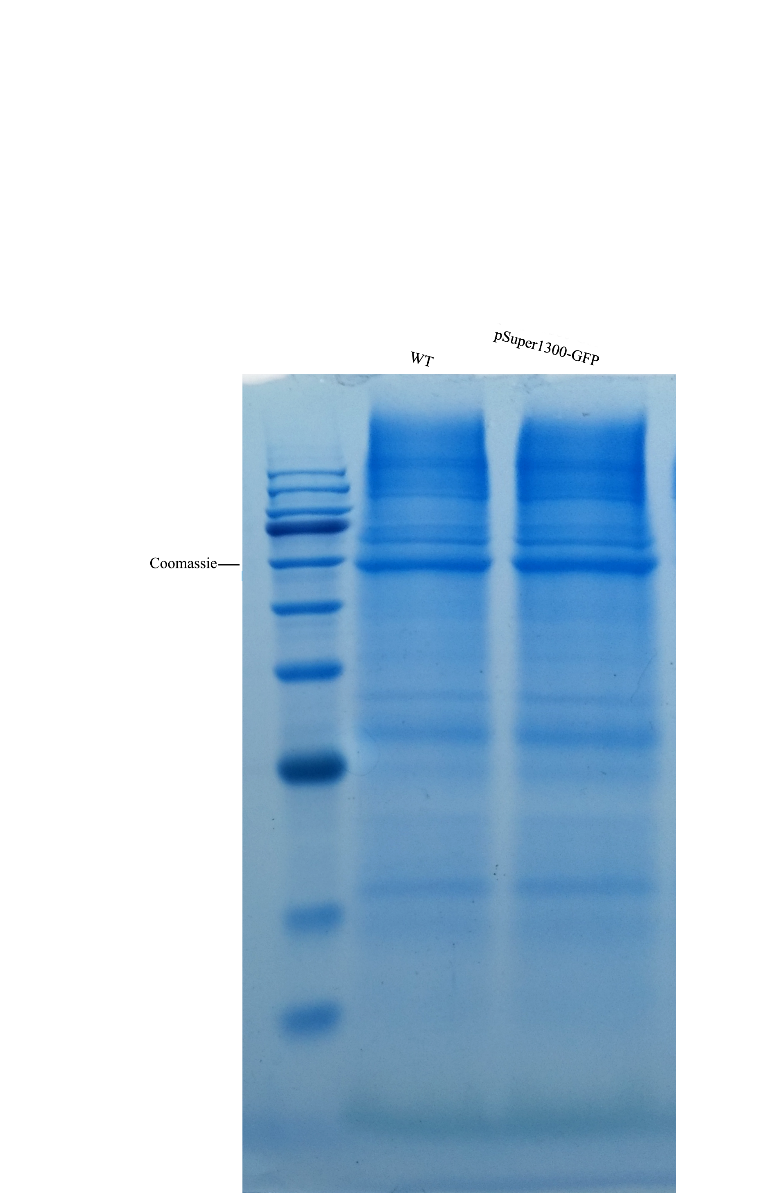


Supplementary Figure 3 Original image of coomassie in wildtype tobacco (WT), tobacco infected with empty carrier (pSuper1300-GFP) showed in Figure 8a


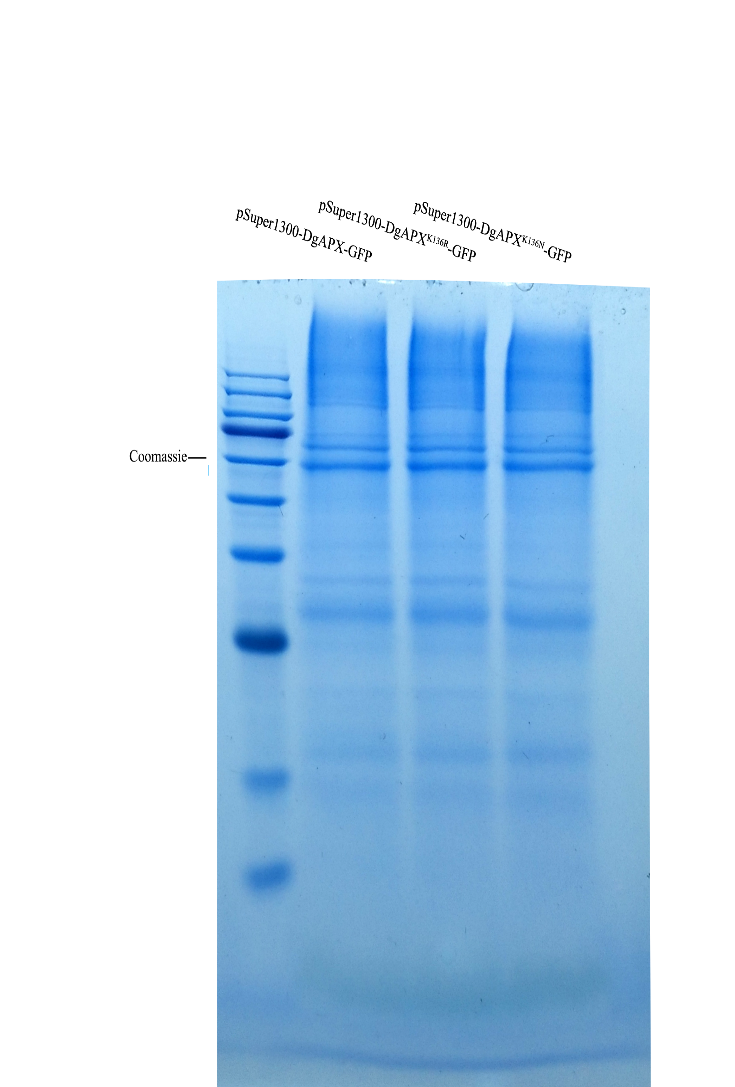


Supplementary Figure 4 Original image of coomassie in tobacco infected unmutated tobacco (pSuper1300-DgAPX-GFP), infected simulant decrotonylation tobacco (pSuper1300-DgAPXK136R-GFP), and infected simulant complete crotonylation tobacco (pSuper1300-DgAPXK136N-GFP) showed in Figure 8a


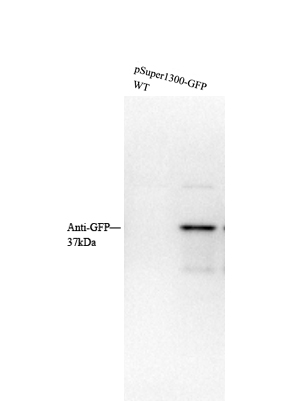


Supplementary Figure 5 Original image of western blot in wildtype tobacco (WT), tobacco infected with empty carrier (pSuper1300-GFP) showed in Figure 8a


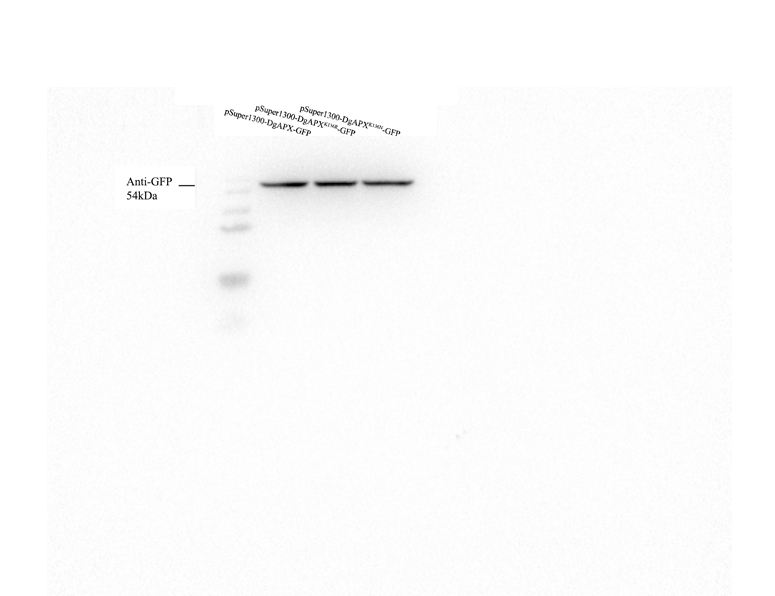


Supplementary Figure 6 Original image of western blot in tobacco infected unmutated tobacco (pSuper1300-DgAPX-GFP), infected simulant decrotonylation tobacco (pSuper1300-DgAPXK136R-GFP), and infected simulant complete crotonylation tobacco (pSuper1300-DgAPXK136N-GFP) showed in Figure 8a
